# Emission Inventories and Particulate Matter Air Quality Modeling over the Pearl River Delta Region

**DOI:** 10.3390/ijerph18084155

**Published:** 2021-04-14

**Authors:** Diogo Lopes, Joana Ferreira, Ka In Hoi, Ka-Veng Yuen, Kai Meng Mok, Ana I. Miranda

**Affiliations:** 1 Centre for Environmental and Marine Studies (CESAM), Department of Environment and Planning, University of Aveiro, 3810-193 Aveiro, Portugal; jferreira@ua.pt (J.F.); miranda@ua.pt (A.I.M.); 2 Department of Civil and Environmental Engineering, Faculty of Science and Technology, University of Macau, Macau, China; kihoi@um.edu.mo (K.I.H.); kvyuen@um.edu.mo (K.-V.Y.); kmmok@um.edu.mo (K.M.M.)

**Keywords:** Pearl River Delta, particulate matter, gridded emission inventories, WRF-CAMx

## Abstract

The Pearl River Delta (PRD) region is located on the southeast coast of mainland China and it is an important economic hub. The high levels of particulate matter (PM) in the atmosphere, however, and poor visibility have become a complex environmental problem for the region. Air quality modeling systems are useful to understand the temporal and spatial distribution of air pollution, making use of atmospheric emission data as inputs. Over the years, several atmospheric emission inventories have been developed for the Asia region. The main purpose of this work is to evaluate the performance of the air quality modeling system for simulating PM concentrations over the PRD using three atmospheric emission inventories (i.e., EDGAR, REAS and MIX) during a winter and a summer period. In general, there is a tendency to underestimate PM levels, but results based on the EDGAR emission inventory show slightly better accuracy. However, improvements in the spatial and temporal disaggregation of emissions are still needed to properly represent PRD air quality. This study’s comparison of the three emission inventories’ data, as well as their PM simulating outcomes, generates recommendations for future improvements to atmospheric emission inventories and our understanding of air pollution problems in the PRD region.

## 1. Introduction

Particulate matter (PM) affects more people than any other air pollutant [1,2] and according to the World Health Organization (WHO) [3], the PM fractions relevant to human health are particles with an aerodynamic equivalent of a diameter less than or equal to 10 µm (PM_10_) and particles with an aerodynamic equivalent of a diameter less than or equal to 2.5 µm (PM_2.5_).

The Pearl River Delta (PRD) region is located in a transitional zone of the East Asian monsoon system. It comprises eleven municipalities; nine are located in mainland China (Guangdong province) and two in the Special Administrative Regions (SAR) of Hong Kong and Macau [4,5]. The PRD has become one of China’s three main economic hubs and it is one of the most densely urbanized regions in the world [6,7]. With rapid development in the region, PM concentration increases along with decreases in visibility have become critical problems [8]. The highest PM levels are observed in the winter months (i.e., December, January and February) when northerly winds bring air pollution from highly polluted areas to the region, and when lower mixing heights and smaller rainfall amounts and frequencies are registered. PM concentrations are lower in the summer months (i.e., June, July and August). In this season, southerly winds from the South China Sea, higher mixing height values and larger rainfall amounts and frequencies favor better air pollution dispersion and deposition [9,10,11].

Aiming to reduce the air pollution problems, the National Ambient Air Quality Standards (NAAQS) of China (GB 3095-2012) were updated by the Ministry of Environmental Protection, adding the PM_2.5_ annual and daily standards. The annual limit values of PM_10_ for special areas such as national parks (i.e., Grade-I standard) and others (i.e., Grade-II standard) were also reduced.

The last report on the progress of the prevention and control of air pollution in Chinese cities showed that air quality had improved when compared with previous years. The PRD registered the lowest concentration levels among all key regions in China (i.e., lower than the Yangtze River Delta and Beijing-Tianjin-Hebei). However, the region still does not fully comply with the NAAQS of China, mainly in the winter when coal-burning and meteorological conditions lead to frequent air pollution episodes [12].

Air quality modeling is a useful approach to better understanding atmospheric pollution patterns in the PRD region. Air pollution emissions are amongst the most important input data required for air quality modeling [13] and several atmospheric emission inventories are available that can be applied over the Asia region.

The Emissions Database for Global Atmospheric Research (EDGAR) is a global emission inventory developed by the European Commission and the Netherlands Environmental Assessment Agency. It provides gridded annual emissions with 0.1 degrees (≈10 km) of horizontal resolution. Anthropogenic emissions of greenhouse gases and air pollutants (namely PM_10_ and PM_2.5_) are calculated by applying a technology-based emission factor approach for domestic sources, road transport, industry and other sectors. Total national emissions by sector are spatially allocated using the location of manufacturing facilities, road networks, land use, and human and animal population densities [14].

The MIX Asia emission inventory was created using a combination of different regional atmospheric emission inventories [15,16,17,18,19,20], applying a mosaic approach. It includes monthly gridded emissions (at a 0.25-degree horizontal resolution) for the years 2008 and 2010 over Asia. These are aggregated into five activities: domestic, road transport, industry, power and agriculture. The emissions include gaseous pollutants, aerosols (i.e., PM_10_, PM_2.5_, organic carbon (OC) and black carbon (BC)), and speciated emissions for the SAPRC-99 (State Air Pollution Research Center 1999 version) and CB05 (Carbon Bond 5) chemical mechanisms [21]. Data on the locations of large emission sources, population densities, road networks and land use are used as spatial proxies to derive gridded emissions from each regional atmospheric emission inventory.

Kurokawa et al. [16] developed the Regional Emission inventory in Asia (REAS) emission inventory. It includes the major air pollutants (i.e., PM_10_ and PM_2.5_) and greenhouse gases over the Asia region. Monthly gridded emissions with a horizontal resolution of 0.25 × 0.25 degrees are provided for domestic sources, road transport, industry and other sectors. Atmospheric emissions are spatially disaggregated using population data, large point source locations, land cover data and road networks [16].

The main purpose of this work is to analyze the air quality modeling performance for PM_10_ and PM_2.5_ concentrations over the PRD, testing different atmospheric emission inventories. EDGAR, MIX and REAS are used to generate temporally and spatially disaggregated emissions as inputs for the air quality modeling system. The atmospheric emission inventories are tested in January (winter period) and July (summer period) of 2014 when the highest and lowest PM levels are recorded, respectively [10]. This year is selected based on its representativeness of the meteorological conditions that are generally observed over the study area and the availability of measured air quality data [10]. 

The paper is organized as follows. In Section 2, the air quality modeling setup and configuration are described. Section 3 presents and discusses the spatial and temporal disaggregation of the emission inventories. Section 4 concerns the system performance of the selected emission inventories. Finally, in Section 5, the main conclusions are presented.

## 2. Modeling Setup and Configuration

**Figure 1:**
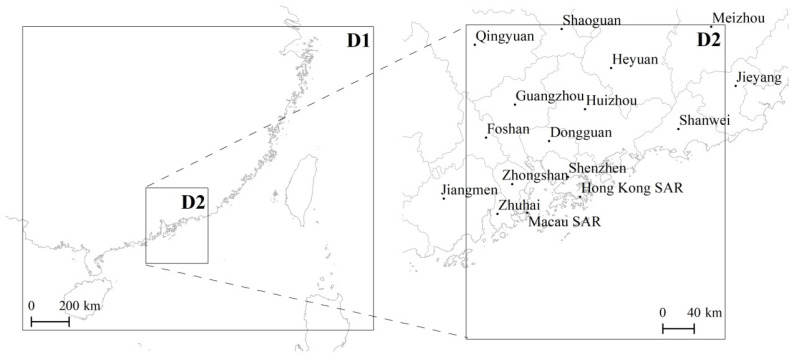
Simulation domains used by the WRF-CAMx (parent grid—D1, 9 × 9 km^2^ resolution; nested domain—D2, 3 × 3 km^2^ resolution) and the cities/regions in the innermost domain.

The air quality modeling system, composed of the Advanced Research Weather and Forecasting Model (WRF-ARW) [22] and the Comprehensive Air Quality Model with extensions (CAMx) [23] was used to simulate the air pollution levels over the PRD region. This air quality modeling system was selected since it has been extensively tested and shown to produce robust and realistic results [24,25,26]. Figure 1 shows the simulation domains used by the WRF-CAMx system and the cities/regions located over the innermost domain are identified.

The WRF-ARW (version 3.8) (National Center for Atmospheric Research, Boulder, CO, USA) [22] domains’ configuration consists of four nested grids: the larger domain comprises the major part of Asia with a grid spacing of 81 × 81 km^2^; the following domain, with a horizontal resolution of 27 × 27 km^2^, covers south-eastern China, the Korean Peninsula and part of south-eastern Asia; the last two domains cover the southeast coast of China and PRD region, with a horizontal resolution of 9 × 9 km^2^ and 3 × 3 km^2^, respectively. This model was initialized with global fields from the National Centre for Environmental Prediction, with a 1° by 1° spatial resolution and a temporal resolution of 6 h. The WRF-ARW input dataset provided information on topographies and land use/land cover data for each domain. The WRF-ARW for three different combinations of parameterization schemes for winter (i.e., January) and summer (i.e., July) was tested [27] and the following configuration was selected as the best set of parameterization schemes: the WRF Single-Moment 6-class scheme [28], the rapid radiative transfer model [29], Dudhia [30], the Monin-Obukhov similarity scheme [31,32,33], the Noah land surface model [34], the Kain-Fritsch scheme [35] and the Yonsei University scheme [36]. The same parameterization schemes were used for all WRF-ARW domains. For more information on the WRF evaluation performance for the PRD region during the simulation periods of this study (January and July), see Lopes et al. [27].

The CAMx (version 6.40) model (ENVIRON, Novato, CA, USA) simulates the atmospheric emissions, air pollution dispersion, chemical reactions and removal of pollutants in the troposphere by solving the Eulerian continuity equation for each chemical species in a system of nested three dimensional (3D) grids [23]. It is suitable for application to different scales ranging from global to sub-urban regions. In this study, the CAMx model was applied to two smaller WRF-ARW domains: a coarse domain covering the southeast coast of mainland China with 9 × 9 km^2^ of horizontal resolution (D1) and a nested domain comprising the PRD region with a grid spacing of 3 × 3 km^2^ (D2). Vertical and horizontal transport/advection were performed using the piecewise parabolic method and an implicit backward-Euler integration scheme [37]. Turbulent diffusion and chemistry were calculated using standard “K-theory” and an Euler backward iterative method, respectively. The surface ultraviolet albedo and surface resistance values for the dry deposition calculations, as well as the seasonal default surface roughness lengths and leaf area index values, were taken from the WESELY89 dry deposition option [23]. The Carbon Bond 6 (CB06) gas-phase mechanism was selected in this study. For PM, the CAMx aerosol scheme splits the particle size distribution into coarse and fine modes. Primary species are simulated as fine and/or coarse particles, while all chemically-formed compounds are modeled as fine particles only.

The global chemical MOZART model (National Center for Atmospheric Research, Colorado, United States of America) outputs every 6 h [38] were used to provide the initial and boundary conditions for the air quality model. Ozone column data were collected from the Total Ozone Mapping Spectrometer (TOMS) dataset [39]. The global inventory EDGAR [14], and the Asian inventories REAS [16] and MIX [21] were chosen to be tested with the air quality modeling system.

## 3. The Atmospheric Emission Inventories

This section presents the methodology used to generate temporally and spatially disaggregated emissions for the WRF-CAMx system. Results over the PRD study area from this disaggregation are also analyzed in more detail with further information regarding the total and spatial distributions of emissions.

### 3.1. Temporal and Spatial Disaggregation

To perform this work, data from the EDGAR, REAS and MIX emission inventories for the years 2008, 2008 and 2010, respectively, were used. The EDGAR, REAS and MIX emission inventories were adapted to generate temporal and spatial emissions for the WRF-CAMx domains. The daily profile for the domestic sector was based on the authors’ lived experiences in Chinese cities. Wang et al. [40] used the same methodology and obtained a similar temporal profile for the domestic sector. For road transport, temporal profiles were derived from the hourly variation of carbon monoxide (CO) concentrations at four roadside air quality monitoring stations (one in Macau SAR and three in Hong Kong SAR). These air quality data were obtained from the Macau Meteorological and Geophysical Bureau (SMG) and the Hong Kong Environmental Protection Bureau (HKEPD), respectively [41,42]. Li et al. [43] compared the CO concentrations measured at the roadside air quality monitoring station in the Macau SAR with the traffic volumes of several nearby roads. The results showed a high correlation between CO and road traffic volume, meaning that the temporal concentration variation of this pollutant could be used as the surrogate of the traffic volume. Zhang et al. [44] further recorded a similar temporal profile using mean hourly traffic counting during weekdays over the Macau SAR. For the other sectors, European temporal profiles were considered [45] due to the limited data available.

With the exceptions of the agricultural and international navigation sectors, the required resolution for the WRF-CAMx domain was obtained using land use and population data. Land use data with 500 m of horizontal resolution was provided by the United States Geological Survey Land Cover Institute [46]. For mainland China, the spatial population distribution developed by Fu et al. [47] with 1 × 1 km^2^ of horizontal resolution was used. For the Macau SAR, Hong SAR, Taiwan and the Philippines, a highly resolved spatial population distribution was produced using census [48,49,50,51] and land use datasets [46]. The population was only considered in the urban grids since the highest emissions and the largest numbers of inhabitants are observed in urban areas [52,53,54]. For the agricultural and international navigation sectors, a spatial disaggregation using only land use data was applied [46] and emissions were allocated to cropland grids and the ocean region, respectively.

The chosen chemical mechanism of the CAMx model requires the input of twenty-one gases and seven PM species including inert, organic and inorganic fractions (for detailed information, see [23]). A literature review was conducted and Chinese chemical speciation profiles were developed for road transport [55,56,57], domestic [58] and solvent activity sectors [59]. For the remaining sectors, the SPECIATE 4.4 database from the United States Environmental Protection Agency was used [60]. Taking into consideration that the EDGAR atmospheric emission inventory includes only annual emissions and the EDGAR and the REAS inventories have similar classifications of activities, the REAS inventory was used to create and apply to EDGAR a monthly temporal profile and a spatial speciation profile (by emission sector), over the CAMx modeling domains.

### 3.2. Total and Spatial Distribution Results

**Figure 2:**
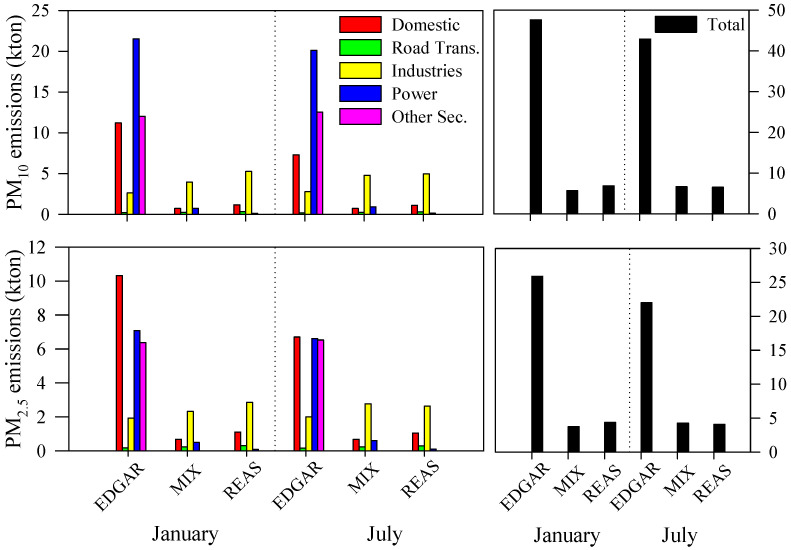
Comparison of each emission inventory’s total emissions (kt) for the D2 modeling domain, by sector and PM fraction, and for the winter and summer months.

Based on the disaggregation methodology, PM emissions for the PRD study area were obtained. Figure 2 shows the PM (i.e., PM_10_ and PM_2.5_) emissions (kt) for each emission inventory by sector, in the winter (January) and summer (July) months, for the innermost WRF-CAMx domain (i.e., D2).

The EDGAR PM_10_ and PM_2.5_ emissions are higher than those from the other two inventories. This is mainly due to other sectors’ activities and domestic activities. For the other sectors, the reason for the large difference is the inclusion in the EDGAR inventory of more emission sources. The other sources in each inventory include the following emission activities: EDGAR) other transport, waste incineration, manure management, manufacturing and construction; MIX) this inventory does not include activities in this emission sector category; REAS) international navigation and other transport. For domestic sources, this is related to the approach used by EDGAR to spatially allocate the total national emissions. Because of the domestic sector’s contribution to the EDGAR inventory, it provides the highest total emissions during the winter when coal-burning is used for domestic heating. The industrial sector’s emissions are similar for the three emission inventories, with slightly higher values estimated by the REAS and MIX inventories. Moreover, the inventories also present similar total emissions for road transport, which is the smaller contributing sector. Besides, the atmospheric emissions provided by EDGAR, REAS and MIX are comparable with the values computed by other studies. For example, Zheng et al. [61] obtained PM_10_ and PM_2.5_ emissions of 687 kt (monthly average of about 57 kt) and 473 kt (monthly average of about 39 kt) for the year 2010 over Guangdong. This province includes Zhaoqing (not considered in this study), Guangzhou, Huizhou, Dongguan, Shenzhen, Zhuhai, Zhongshan and Foshan.

**Figure 3:**
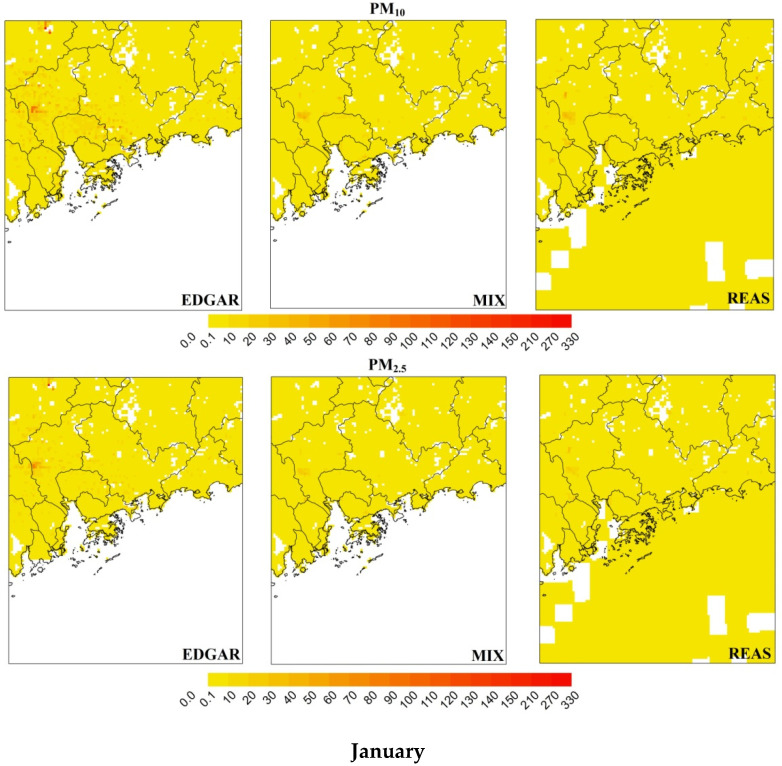

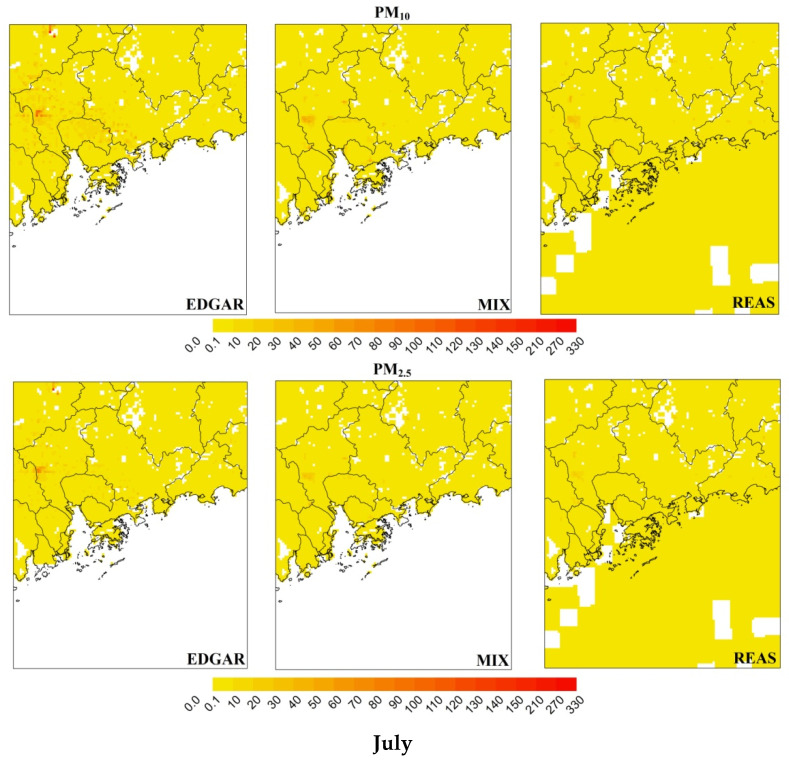
Spatial distribution of PM_10_ and PM_2.5_ emissions (t) over D2 (3 km^2^ of resolution) in January and July.

The spatial distributions of PM_10_ and PM_2.5_ during the winter and summer months are shown in Figure 3.

The EDGAR, MIX and REAS disaggregated data present the highest PM emissions in the north of Qingyuan (PM_10_ ≈ 300 t and PM_2.5_ ≈ 200 t), over the Macau peninsula (PM_10_ ≈ 70 t and PM_2.5_ ≈ 40 t) and Guangzhou (PM_10_ ≈ 50 t and PM_2.5_ ≈ 40 t), respectively (Figure 1 shows the locations). Focusing on the activity sectors with larger differences among the three inventories (i.e., domestic and other sectors), it is possible to identify for the domestic sector the highest emissions over different areas, for all inventories. For the other sectors, the EDGAR data show emissions mainly over the central region of the PRD (Guangzhou, Dongguan, Foshan and Zhongshan) while the REAS data result in similar values over the entire study area, including emissions over the South China Sea, which are due to international shipping activities. For road transport, emissions are higher over urban areas, and large industrial sector emissions values are estimated in Guangzhou for all of the tested inventories.

In the next section, the WRF-CAMx system’s performance for the winter and summer simulation months is presented using the EDGAR, MIX and REAS inventories.

## 4. WRF-CAMx System Emissions Comparative Performance

**Figure 4:**
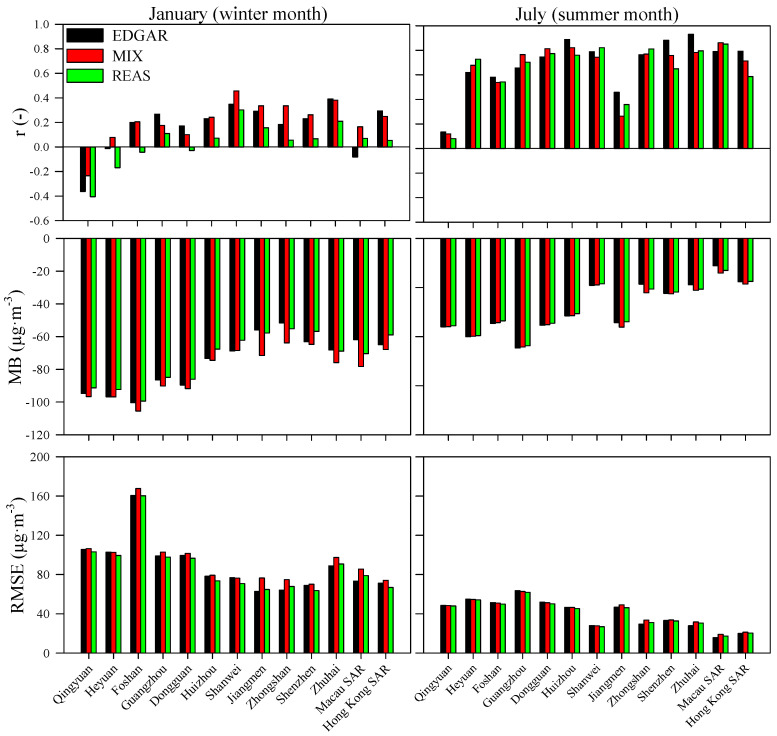
Daily model performance of PM_10_ in winter and summer months.

**Figure 5:**
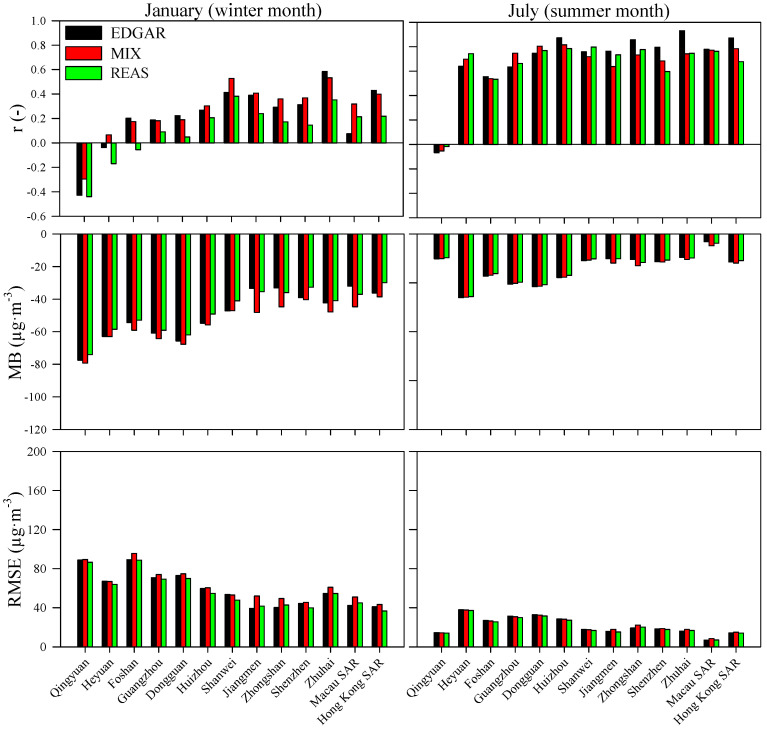
Daily model performance of PM_2.5_ in winter and summer months.

The WRF-CAMx system was applied using the EDGAR, MIX and REAS inventories. The air quality modeling results were compared with data from thirteen air quality monitoring stations over the study region (one site for each city/region). An evaluation of the system’s performance for PM_10_ and PM_2.5_ was based on the methodologies proposed by Heinke and Sokhi [62] and Borrego et al. [63], and the following statistical parameters are presented: correlation coefficient (r), mean bias (MB) and root mean square error (RMSE). Figure 4 and Figure 5 depict comparative statistics between daily WRF-CAMx outputs and daily measured values of PM_10_ and PM_2.5_, respectively.

**Figure 6:**
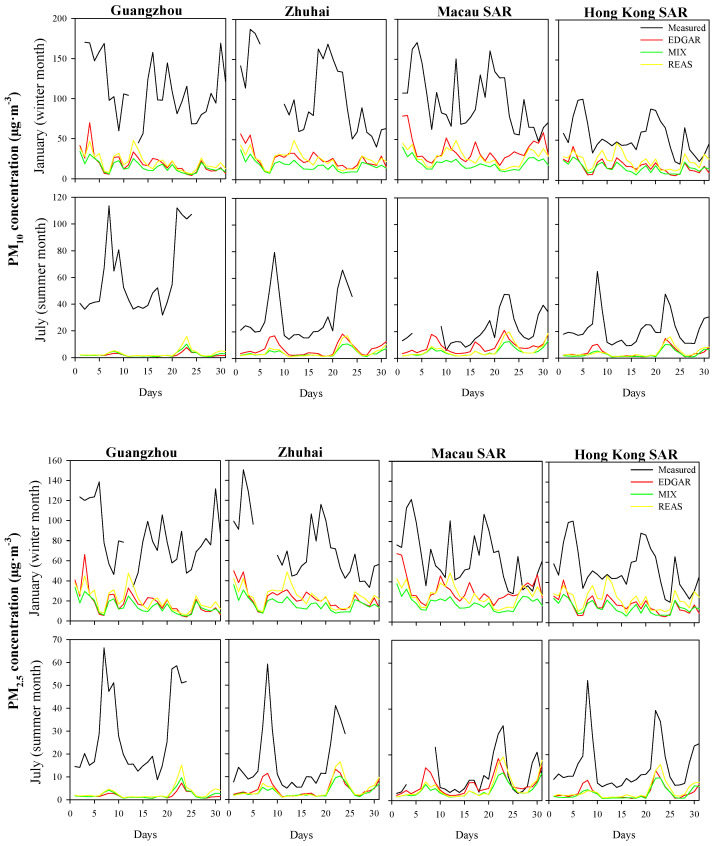
Daily time series of modeled and measured PM_10_ and PM_2.5_ concentrations for four air quality monitoring stations located over D2 in January and July 2014.

For PM_10_, the air quality modeling simulations revealed similar levels of accuracy using the different emission inventories. EDGAR-based results, however, showed a slightly better performance with higher correlation coefficients over the entire simulation domain and lower errors in the southern coastal regions of mainland China. Higher correlations and lower errors were obtained for the summer month when lower air pollution concentrations over the simulation domain were measured. The correlation coefficient ranged from −0.41 to 0.46 (REAS and MIX) in the winter month and 0.08 to 0.93 (REAS and EDGAR) in the summer month. These results are in agreement with previous studies, for example, a correlation coefficient of 0.32 (hourly model performance evaluation) for a summer episode was obtained by [64] for a modeling simulation over the PRD region. [65] calculated a correlation coefficient of 0.47 and 0.57 (daily model performance evaluation) for a winter and summer period, respectively. The current simulations tended to underestimate the PM_10_ levels (negative MB and time series are presented in Figure 6) for both seasons, which is a common behavior for PM_10_ simulations over the study region (e.g., [65,66]). The overestimation of wind speed by the WRF-CAMx system [27] may have contributed to the dispersion of atmospheric pollutants, leading to lower PM_10_ concentrations [67]. The magnitudes of errors ranged from 62.7 to 168 µg·m^−3^ (EDGAR and MIX) in January and 15·8 to 63·5 µg·m^−3^ (EDGAR) in July. These results are in agreement with those obtained by Xun-lai et al. [64] during an autumn episode (RMSE was 36 µg·m^−3^).

Similar to the PM_10_ results, the WRF-CAMx outputs with the EDGAR emission inventory showed a slightly better accuracy for PM_2.5_ simulations, as well (Figure 5). The best PM_2.5_ performance (higher correlation coefficient and lower error) was also found in the summer period. Qin et al. [65] and Kwok et al. [66] observed the same behavior for PM_2.5_ simulations over the study region. The correlation coefficient ranged from −0.44 to 0.58 (REAS and EDGAR) for the winter month and −0.07 to 0.93 (EDGAR and EDGAR) for the summer month. These daily results are similar to those obtained by Qin et al. [65] in the winter (r = 0.60) and summer (r = 0.87), but the results of this work were less satisfactory than those presented by Liu et al. [68], with a correlation coefficient ranging from 0.74 to 0.94. However, these authors obtained this air quality modeling performance for a shorter air pollution episode (24 h). The modeling system tends, as observed for PM_10_, to underestimate the PM_2.5_ levels (negative MB and time series are presented in Figure 6) for both seasons. The RMSE varied from 36.7 to 95.6 µg·m^−3^ (REAS and MIX) and 6.87 to 38.0 µg·m^−3^ (EDGAR) in winter and summer, respectively. Wang et al. [69] obtained an RMSE of 28 µg·m^−3^ in winter and 10 µg·m^−3^ in summer, while Chen et al. [26] recorded an RMSE of 21.75 µg·m^−3^ in winter. Besides, Liu et al. [68] computed an RMSE of between 29.0 and 56.5 µg·m^−3^ for an air pollution episode (24 h) over the PRD.

Figure 6 provides the time series for January and July at four air quality monitoring stations located near the main PM sources (Guangzhou) and the coastline (Zhuhai, Macau SAR and Hong Kong SAR).

Despite an underestimation, globally, WRF-CAMx when using different atmospheric emission inventories presents similar results, and the inventories can capture air pollution’s seasonality (with the highest levels in January and the lowest in July). In the winter season, domestic coal-burning leads to frequent air pollution episodes [12], which are not properly captured by the simulation because this air pollution source over the PRD region is underestimated [66]. In the summer period, the system’s ability to simulate the observed magnitude peaks is lower under the predominant typhoons and Pacific high system weather conditions [70,71]. In the particular simulated 2014 summer month, air pollution episodes were measured by almost all air quality monitoring stations on 7–9 and 21–23 July. In these periods, the region was affected by the approach of typhoons [41]. The air pollution episodes were recorded three days before each typhoon landed. These results are in agreement with previous studies over the Southeast Asian region where, before the arrival of a typhoon, high air pollution levels are observed at the periphery of the typhoon’s affected area due to the impact of downdrafts, atmospheric circulation field distribution and a high/uniform pressure system at the tropical cyclone front [70,72,73]. Under these conditions, it is difficult to obtain good simulation results, whatever emission inventory is used.

**Figure 7:**
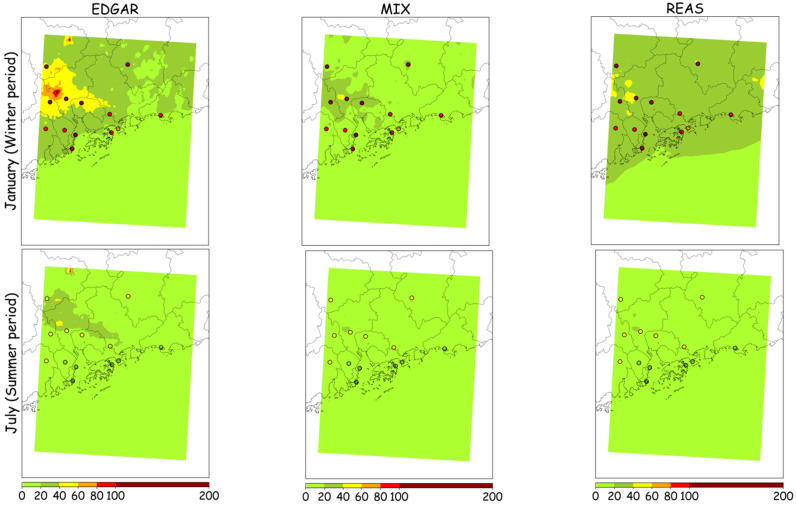
Monthly average PM_10_ concentrations (µg·m^−3^) in the winter and summer months, as modeled by the WRF-CAMx system and measured at the air quality monitoring stations (small circles).

**Figure 8:**
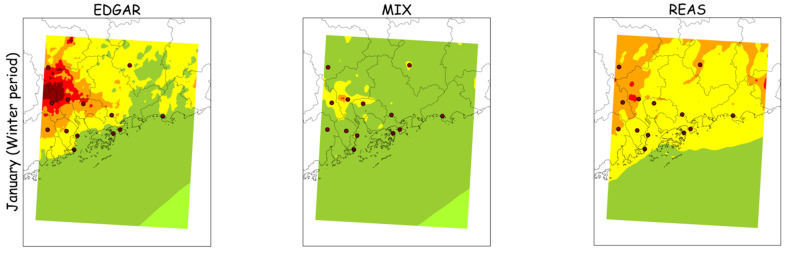

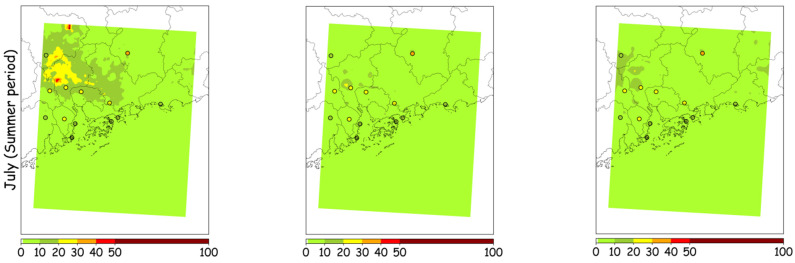
Monthly average PM_2.5_ concentrations (µg·m^−3^) in the winter and summer months, as modeled by the WRF-CAMx system and measured at the air quality monitoring stations (small circles).

Figure 7 and Figure 8 display the spatial distributions of modeled monthly average concentrations of PM_10_ and PM_2.5_, respectively, for the winter (January) and summer months (July) using the EDGAR, MIX and REAS emission inventories. Measured monthly averages for the air quality monitoring stations are also represented by small circles.

Air quality modeling results, when applying EDGAR, REAS or MIX, show different spatial distributions of PM levels, but high values for all tested study cases are estimated over the Guangzhou region. The inventories reproduce reasonably well the air pollution concentrations over the study area in summer but they are less satisfactory in winter. These results may be due to uncertainties in the atmospheric emission inventories, namely the poor representation of local sources’ characteristics and locations or spatial variability of emission values, along with errors in the meteorological simulations related to particular weather events.

## 5. Conclusions

This work aimed to evaluate the WRF-CAMx system’s performance when simulating PM_10_ and PM_2.5_ concentrations over the PRD region using different atmospheric emission inventories (i.e., EDGAR, MIX and REAS). In general, the EDGAR, MIX and REAS emission inventories presented higher atmospheric emissions in the winter than in the summer period. The major differences between them were obtained for the domestic and other sectors. For all remaining activities, the total values and spatial distributions of emissions were similar among the three emission inventories. The PM simulation results with EDGAR showed a slightly better performance. The quality of the results is comparable to other air quality modeling applications over the study region, with an underestimation of PM levels. The system still requires improvements in the atmospheric emission inventories’ disaggregation, focusing on the temporal and spatial distributions of local air emission sources and on the contribution of natural sources, such as wildfires and sea salt biogenic emissions, as well as road dust resuspension.

These results could help to improve atmospheric emissions’ estimation and our understanding of air pollution problems over the PRD region.
